# We need to keep a reproducible trace of facts, predictions, and hypotheses from gene to function in the era of big data

**DOI:** 10.1371/journal.pbio.3000999

**Published:** 2020-11-30

**Authors:** Simon Kasif, Richard J. Roberts

**Affiliations:** 1 Bioinformatics Program, Boston University, Boston, Massachusetts, United States of America; 2 Department of Biomedical Engineering, Boston University, Boston, Massachusetts, United States of America; 3 New England Biolabs, Ipswich, Massachusetts, United States of America

## Abstract

How do we scale biological science to the demand of next generation biology and medicine to keep track of the facts, predictions, and hypotheses? These days, enormous amounts of DNA sequence and other omics data are generated. Since these data contain the blueprint for life, it is imperative that we interpret it accurately. The abundance of DNA is only one part of the challenge. Artificial Intelligence (AI) and network methods routinely build on large screens, single cell technologies, proteomics, and other modalities to infer or predict biological functions and phenotypes associated with proteins, pathways, and organisms. As a first step, how do we systematically trace the provenance of knowledge from experimental ground truth to gene function predictions and annotations? Here, we review the main challenges in tracking the evolution of biological knowledge and propose several specific solutions to provenance and computational tracing of evidence in functional linkage networks.

## Introduction

Biological research has generated many foundational and translational discoveries with profound impacts on health, wellness, biotechnology, and our scientific understanding of living organisms. However, recent technological developments have challenged even the best scientists in the largest centers to keep pace with the avalanche of data, predictions, and papers produced by high-throughput technologies, big computing that speeds up analyses coupled with AI-driven interpretations, and the sheer size of the community using these technologies. This landscape is even more challenging for small laboratories and has forced many laboratories (big or small) to become more narrowly specialized in order to produce scholarly and reproducible work at a time when reproducibility has become an urgent concern. While some argue reproducibility problems have grown severe enough to produce a crisis, there’s no debate that the great magnitude of data, analyses, predictions, and papers will only increase and potentially overwhelm the community in the future. As Charles Darwin once said:

“False facts are highly injurious to the progress of science, for they often endure long; but false views, if supported by some evidence, do little harm, for everyone takes a salutary pleasure in proving their falseness.”

In times past, the interpretation of novel DNA sequence depended almost completely on experimental validation of the proteins encoded and direct tests of either biochemical or biological function. This, combined with genetic analysis, then enabled accurate descriptions of function. However, because of the vast amount of DNA sequence now available, we no longer have the time or luxury of experimentally validating function. We instead infer it from what is known more generally about the function of related proteins in other, often model, organisms. Usually, this is carried out by computational comparison and analysis and the concomitant transfer of functional annotation using evolutionary, machine learning, or network analysis.

In the simplest case, consider that protein A is annotated with a function and protein B is 95% identical, then we can feel somewhat confident that the 2 proteins have the same function, unless we have prior knowledge that just 1 or 2 changes to its sequence can alter function. If protein A is an experimentally characterized protein, with a well-defined biochemical function, then our inferences about protein B are on relatively solid ground. But what if the annotation of protein A is itself based only on computational similarity to another protein? Then our confidence in the annotation should be diminished for as one continues down the stream of inferences, they inevitably become incorrect. We see this happening routinely in sequence databases. What kind of evidence should be kept to document the “semantic path” between the newly annotated gene and the experimentally tested protein? Clearly, we need trustworthy links to evidence that are sufficient to either explain or question the validity of the new annotation.

The issues are equally applicable to the increasing number of systems that associate human genes with phenotypes such as essentiality, aging, or disease risk [1]. In these systems, the initial experimental or clinical phenotype labels are obtained by genetic experimentation in model organisms or from Online Mendelian Inheritance in Man (OMIM) for disease risk in human populations. Then, these phenotype labels are propagated to other genes in functional linkage networks and are combined with genetic data obtained from large genome-wide association studies (GWAS) studies to prioritize follow-ups. Relevant phenotype predictions and GWAS scores are commonly integrated to elevate the priority of a follow-up study of a gene associated with disease risk. Thus, provenance and transparency are equally desirable traits of prediction algorithms that are used to prioritize human genes in resource-intensive GWAS studies and their detailed and costly follow-ups by the biology community [1,2].

## Few predictions have a provenance trace to experimental sources

We will first document our own experience and a startling observation we made in the project COMBREX (Computational Bridges to Experiments) [3]. Consider the “simplest” of all biological questions. What does a gene do in a cell, or more specifically, what is the biochemical function of the protein that the gene codes? COMBREX discovered that a tiny fraction (roughly 1%) of bacterial function assignments to genes in microbial organisms are supported by experimental evidence that can be explicitly and robustly traced to a publication [1]. But if we do not have the provenance to the paper where the experiment was conducted, how do we decode the context of the experiment that includes the organism, the biological conditions (e.g., antibiotic stress or oxidative stress), the experimental platform, or even the exact DNA sequence used in the experiment? Without provenance and a trace to experimental sources, how can we be sure of the validity of functional annotation in current databases? However, virtually none of the predictive methods used in AI or Machine Learning (ML)produce an automatic trace to evidence.

The early version of COMBREX [3] implemented a first-of-a-kind straw man heuristic algorithm to provide a **provenance link** from a predicted annotation to its experimental source. The simplest provenance prediction algorithm is based on similarity between proteins. COMBREX, therefore, attempted to identify a similar protein with solid experimental evidence stored in a Gold Standard Database that agrees with a biochemical function prediction. It is not always possible to do. When it is possible, these links to evidence may not be sufficient to prove the function of the new protein. However, it is useful to at least initiate the process of evidence tracking to motivate and inform subsequent improvements. This COMBREX method and many other simple heuristics can be used to construct putative evidence paths to experimental knowledge [4]. We discuss more rigorous methods below for tracing predictions to experimental sources in network based function prediction.

## Network-based functional annotation: High popularity and low interpretability

The annotation problems are further complicated by the lack of transparency through which functional labels are produced by network-based computational predictions. The community is increasingly using **guilt by association** in networks to assign hierarchical function descriptors to proteins (e.g., Enzyme Commission (EC) numbers or GO labels), but these guilt propagation methods are becoming increasingly complex and opaque. This multifaceted story continues to grow in complexity as the gene function annotation community increasingly uses **network propagation** techniques that we and others popularized in biology almost 20 years ago [5–7]. These belief and knowledge propagation methods deploy complex functional linkage networks that we refer to as knowledge networks (KNs). These knowledge networks link facts, predictions, and hypotheses to enable further belief or knowledge propagation [8]. Belief propagation has a specific technical meaning in causal networks, whereas label propagation can take many mathematical forms and algorithmic implementations. Thus, we use the informal term of KNs to capture the use of networks to propagate beliefs and knowledge. In these KNs, a protein may be hypothetically linked to many proteins (neighbors in the network), and each neighbor is linked to many others with multiple functional assignments [1,2,9,10]. When a prediction is made computationally by “belief, label, or knowledge propagation” from the entire network, what are the most important sources of evidence that contributed to this new prediction?

**Fig 1:**
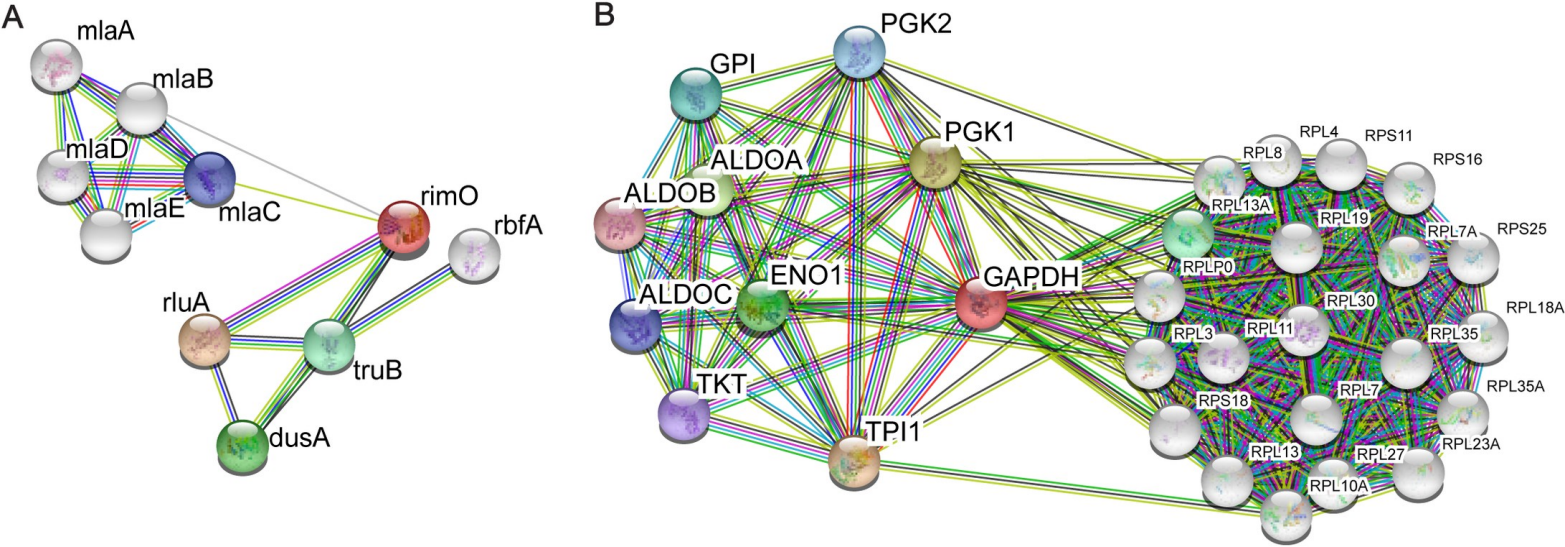
Functional linkage networks in bacteria and human genomes. The networks are produced by the STRING database. Functional linkage relationships could be based on homology, coevolution, genomic context, protein–protein interaction screens, co-expression in a set of experiments, and other correlative or experimental evidence and became standard representations in computational biology. Each node has an associated functional label produced either by experiment or prediction. STRING, Search Tool for the Retrieval of Interacting Genes/Proteins.

In Fig 1, we first show 2 examples of the intuitive and commonly used language of functional linkage graphs to display functional relationships between genes and the challenges of network-based function prediction. We use the widely used Search Tool for the Retrieval of Interacting Genes/Proteins (STRING) database to illustrate 2 important special cases of the challenges of **functional annotation** in functional linkage graphs. In Fig 1A, we document our own experience correcting the functional annotation of the gene rimO in *Escherichia coli*. This gene was originally annotated as an RNA methyltransferase based on homology to miaB and was called miaB-like methyltransferase. We later established experimentally that this gene is in fact a protein methylthiotransferase that modifies a residue in a ribosomal protein S12, which is conserved from bacteria to human [11].

In Fig 1B, we describe the functional linkage of glyceraldehyde 3-phosphate dehydrogenase (GAPDH), a well-known **moonlighting** protein with a variety of biochemical and biological functions. Such proteins pose more significant challenges for annotation that we believe can be resolved by proper provenance links and enforcing consistency constraints during traceable computational or manual annotation.

Our main concern is that automated annotation systems are now assigning functions to genes based on complex learning and propagation algorithms in noisy knowledge networks making the provenance of a prediction even less traceable or transparent [6]. The predictions most notably lack an evidential trail back to a protein of known function or the relevant experimental data informing consequent annotations. Complex network propagation algorithms are now widely used for phenotype prediction and drug repositioning or discovery studies with significant medical and economic impact on future medical and biotechnology development [1,2,12–14]. They are also used to identify new clinical targets. The lack of traceability, or rigorous provenance, can lead to circular logic and self-incriminating paths of evidence, as gene functions are propagated and then “statistically validated” using leaky training–testing processes. That is, the annotation in testing sets used for validation is commonly obtained by machine learning algorithms that previously propagated functional or phenotypic labels from subsets of the training sets to statistically validate their predictions without tracking paths to provenance. In other words, for each prediction, what is the trace to experimentally determined evidence via computationally propagated beliefs and annotation labels?

## Provenance tracing to experimental sources in functional linkage networks

**Fig 2:**
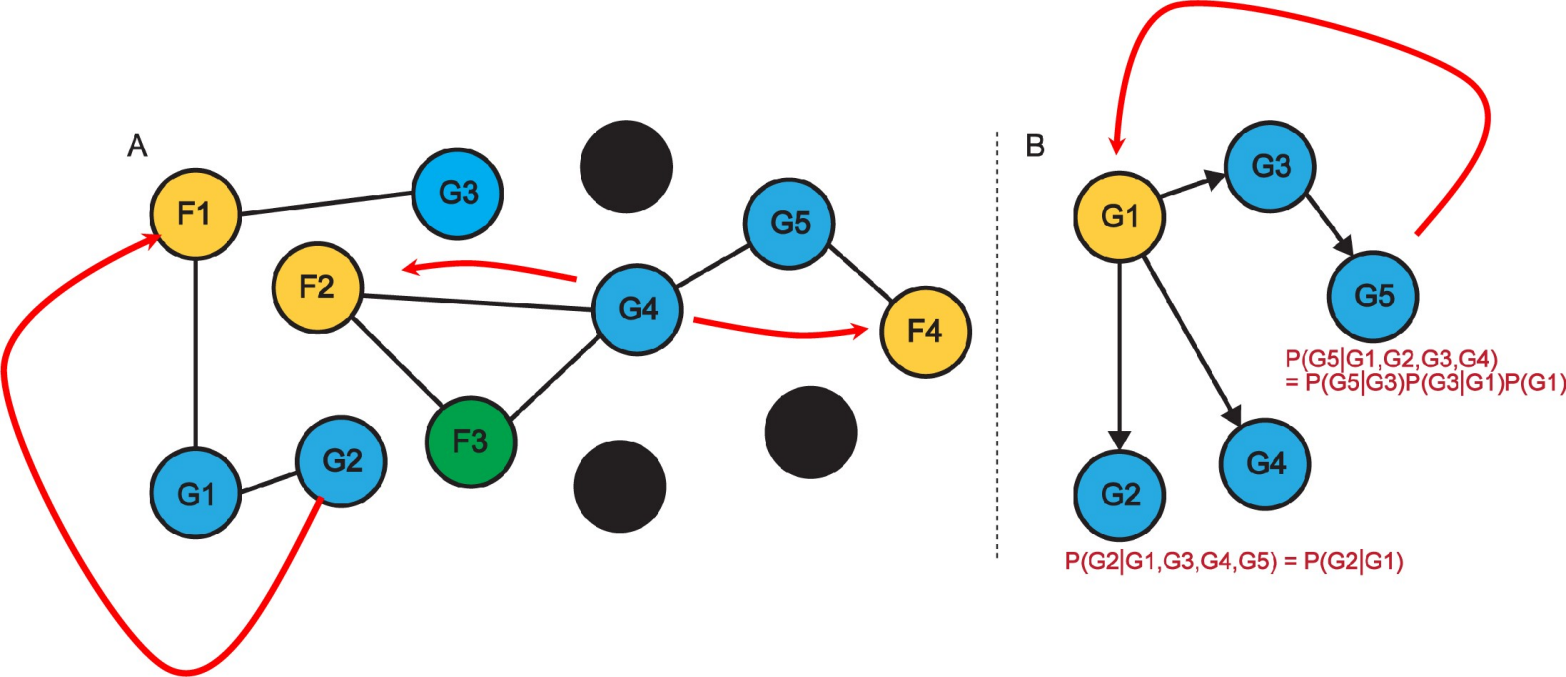
Tracing provenance in functional linkage graphs (2A) and causal networks (2B). The nodes correspond to genes, and the colors are adopted from COMBREX. This coloring scheme was inspired by skiing signs (green, blue, black slopes, and an additional color of gold). Black labels correspond to genes that have no current predictions and therefore are most challenging to experimentally test. Green and gold genes have experimental evidence, and blue genes have predictions. Green labels suggest that some experimental evidence might be missing such as the organisms used or conditions under which the experiment was performed. All blue nodes should ideally have a provenance link to a gene that may demonstrate a phenotype associated with this gene in a genetic experiment or its biochemical function.

There are many heuristic ideas for tracing provenance in networks. We document a simple heuristic method for network diffusion–based prediction and additionally propose a new methodological idea: using causal inference to trace provenance and evolution of biological knowledge in networks used for gene function prediction. There is a real opportunity to approach these challenges systematically using causal inference, graphical models, and related techniques [8,15]. We intuitively sketch out the intuition in Fig 2B. The detailed algorithms require technical developments beyond the scope of this essay.

The causal analysis research area is developing rapidly and generating widely available and readily usable open access software. We suggest that tracing provenance can be implemented using systematic causal inference frameworks [15–17] in addition to the heuristic solutions. Why is causal inference applicable here? Consider the case where gene G1 was experimentally annotated with a functional label that was subsequently propagated to genes G2, G3, and G4 from G1 (Fig 2B). The experimental evidence can be based on biochemical assays or perturbations. The experimental evidence is only available for G1, which is part of the Standard Database. More formally, our belief in the functional annotation of gene G2 given annotations for genes G1, G3, G4, and G5 is conditionally independent of the beliefs associated with G3, G4, and G5 given our knowledge about G1 (i.e., P(G2| G1, G3, G4, G5) = P(G2 | G1)). In this case, we can clearly report G1 as the sole provenance for G2. This is a highly simplified scenario. Often, multiple papers, experiments, and genes are needed to establish a provenance for a functional label and to warrant its inclusion in the Gold Standard Database.

We note that a special case of causal tracing to experimental sources can be performed by phylogenetic inference. For instance, an experimentally determined functional label in 1 organism is propagated to the ancestor of the protein in a phylogenetic tree and then can be properly traced through orthology downwards to predicted annotations. Thus, our causal annotation proposal generalizes the traditional tracing of evolutionary relationship in phylogenetic trees to the much broader framework of functional linkage graphs where the functional inference is much more general and complex. There are many languages capturing causal representations across data sciences going back to Sewell Wright’s seminal work on path analysis in genetics. We suggest using modern AI-driven causality frameworks as a useful point for building a probabilistic foundation of provenance for biological science.

Fig 2A shows that the annotation of genes G1 and G3 can both be directly traced to the functional label F1. Gene G2 requires a longer provenance trace via G1 (illustrated with a red connector). We cannot annotate G2 just with a provenance link to F1 because if G1 is tested and results in a different function, the provenance link is no longer valid. In fact, G4 is linked directly to 2 proteins with 2 different functions F2 and F3. Both links can be stored. However, G4 is also indirectly linked to F4 via G5. This creates a potentially multifunctional assignment to G4 with multiple provenance links. Resolving this type of conflict can be theoretically done by network-based function prediction used in the field. [8].

In Fig 2B, we exemplify the concept of **“causal annotation”** using Bayes Networks, a popular AI platform [18]. The annotation of G5 is formally requiring a full probabilistic trace to G1 via G3, but it is conditionally independent of the functional label assigned to G2 and G4. The red curve traces the provenance of G5 to G1.

We emphasize the sensitivity of provenance to the topology of the functional linkage network and the inference method. However, rudimentary provenance tracing in functional linkage networks is within reach for current AI-based methods.

## Provenance to experimental sources with network diffusion

Network diffusion is a method developed in computer science (CS) and AI and ported to biology to propagate labels. Network diffusion from a node V1 to another node V2 roughly captures the probability that a random walk starting at V1 will reach V2 after a designated number of steps. Many authors use heat diffusion from a node to other parts of the graph, smoothing the heat from a few high heat sources over the entire network to achieve better consistency of label assignments to network neighborhoods. Network diffusion has become a common method for function label propagation in biological networks popularized in part by the success of Google’s PageRank algorithm to identify relevant pages. It generalizes the simpleminded guilt by association from neighbors to propagating labels to nodes that absorb high diffusion from heat from multiple nodes with experimentally confirmed function labels. We note that each diffusion score for a predicted label in a typical case is just a linear sum of diffusions from all nodes with experimental evidence. That is, the diffusion score Si = Sum Kij from all nodes Vj with experimental evidence where Kij is the diffusion from Vj to Vi.

We observe that, in Fig 2, the diffusion heat obtained from F4 at G4 would be stronger than the diffusion heat propagated from either F2 or F3. This is perhaps counterintuitive given that the network proximity from F2 and F3 is shorter (1 link). But diffusion heat from F2 is diffused to 2 nodes and therefore is reduced. The system may then predict the function of G4 to be F4 and produce the appropriate provenance links. Alternatively, if all diffusions are higher than expected by chance, the system may produce 3 annotations with 3 separate sources.

For network diffusion algorithms [2,7] that propagate functional labels, we can devise a very simple trick to “trace” predictions. Since the prediction is made by a diffusion from all experimental sources, the provenance tracing system can compute a significance associated with each coefficient Kij (similar conceptually to what is done in regression). This technique can associate a statistical significance of the contribution from each experimental or computational annotation to the final score used to make a gene function prediction. The system then may only keep links to the genes that pass a significance threshold. We observe that this simple trick can be used for any machine learning methods that use a linear combination of evidence to make predictions such as logistic regression or 1-layer neural networks. Prize collecting, Steiner tree methods in networks can also be deployed as another long-term heuristic connecting experimental evidence with predictions.

## Semantic extensions

The provenance tracing idea can be naturally expanded to more complex representation of protein function. Consider a framework where each gene is a node in a large causal network capturing our knowledge about all genes in a given set. At the moment, this annotation is typically just a simple functional label such as “receptor kinase” or “DNA-binding protein” or a pointer to an ontology. In the future, protein function annotation can be extended to text, mechanisms, logical formulas, or a model. For instance, the bioinformatics community already built some tools for parsing text descriptions of protein function and converting those into a structured annotation such as EC numbers. The temporal dimension in building experimental knowledge (e.g., year of publication and/or citations) and improvements in natural language processing allow us to assign directed or undirected arcs to genes (and papers) documenting putative paths of “moving” annotation or evidence from 1 study to another. In some cases, functional hypotheses can be proven as facts by both prior publications and future work, often requiring both. If we had a formal structural representation of knowledge networks (e.g., as a graphical model), we could apply causal inference to identify the key contributors to knowledge or beliefs. The advent of genome scale perturbations [19] is increasing the opportunistic benefit of tracking provenance using logical, graphical, or causal frameworks as well because the observed phenotypes can be propagated and traced reproducibly and reliably.

## Conducting experiments on knowledge gaps

Moreover, KN platforms complemented with provenance can be used to identify knowledge gaps [20–22] and cost-effective ways to close them. There are several documented systematic ways to track, predict, and identify gaps in growing knowledge captured by graphical models and networks. Consider a protein has multiple domains and all are required to perform a function F (e.g., a DNA methyltransferase requires a DNA-binding domain, a recognition domain, and a catalytic domain). All 3 must be documented with appropriate experimental evidence to form a cohesive functional description of the protein as a whole. This is of special importance as synthetic biology generates many creative variants of proteins from fusion of components found in different proteins [21]. We can still derive much insight by framing this kind of knowledge in the language of causal networks (or related representations). For instance, active learning algorithms in causal networks can be used to compute the minimal or most cost-effective set of proteins (or other hypotheses) that can be tested experimentally to produce a significant decrease in uncertainty in the network and close the knowledge gaps more efficiently [15–17].

This approach enables another desirable opportunity to advance knowledge as we advocated for in COMBREX and follow-up white papers. We proposed augmenting human intuition by driving experimental research toward closing knowledge gaps in protein function space in the most cost-effective fashion [3]. This can be done by prioritizing the most informative experiments that can best reduce uncertainty in the knowledge (belief) network. Formally, such experiments produce the highest information gain in causal networks. In some cases, these experiments may be associated with nodes that produce the highest diffusion in networks. These ideas are generally based on Active Learning frameworks in AI. Active Learning has been independently proposed for different biological or scientific applications [21].

## Call for action: Provenance tracing to experimental sources as more reliable foundation for systems and synthetic biology

We call for agencies and foundations to increase effort in the area of computational provenance tracing. These ideas complement the existing reproducibility efforts in statistics (e.g., R) and software engineering (e.g., GALAXY). We see computational provenance in KNs as an essential requirement for developers of future Science Informatics systems and more broadly Data Science used in Scientific Discovery.

In this paper, we focused on broadly applicable solutions to tracing provenance in network-based function prediction systems. However, similar issues apply to predictions made by more traditional machine learning systems (such as decision trees or logistic regression) and even more so for predictions made by deep learning architectures. For logistic regression type predictions, the general concept we outlined above would work. Decision trees may select the most informative features automatically, and these informative features just need to be recorded in the knowledge base (for each protein separately) to provide a provenance trace, which is not done now, but should be. Deep nets remain a big challenge, and it is very difficult to produce an interpretable prediction. However, our proposed solutions would cover a large and widely used set of function prediction methods.

We note that the database community independently studies important provenance questions, but the semantics of provenance in relational databases or software engineering is completely different from our proposal to trace predictions to experimental evidence. In integrated or distributed databases, provenance means which specific database did an entry originally belong to when we integrated multiple data files. Clearly, it is an important practical issue as well but very different in scope or techniques to the trace of provenance needed to establish evidence for a predicted annotation.

This essay independently suggests an urgent need to generate a Gold Standard Database of functional annotations for the Human Genome and other key model organisms. If we cannot trace the provenance of a functional annotation currently assigned to a human gene, how can we be confident of its precise functionality? Given the available annotation, it will not take a massive effort to validate all high-priority genes that can be used as a platform for propagating and judiciously providing provenance to predicted functional annotation.

We would like to emphasize that our proposals should not be seen as a critique of the seminal work and research performed by world-class annotation centers and databases such as National Center for Biotechnology Information (NCBI), Gene Ontology, Flybase, BioCyc, STRING, GeneCards, and many others. We are deeply grateful to the biological database community for enabling and supporting biomedical science. In addition, we are also profoundly grateful to the transformative work in protein function prediction that includes many ground-breaking algorithms and systems. We want to point out the challenges facing these efforts, however, and propose to start an active community dialogue to produce novel solutions. These solutions may include our specific proposals described above or others.

If we are ever to perform synthetic biology [23,24] in a meaningful manner, we need to know the functions of the genes already present in the organism we aim to modify. The prospect of unanticipated consequences due to unforeseen interactions is a risk whenever we introduce new genes or replace old ones. While such risks can usually be overcome, they inevitably hinder efforts due to trial and error when pursuing synthetic biology approaches. The more we know about the functions inherent in an organism, the more precise we can be in engineering new traits. Thus, a clearly traceable knowledge of existing gene function can provide a sound foundation upon which synthetic biology and systems biology efforts, together with AI methods, can be expected to drive and expand our understanding of biology.

## Abbreviations

AI: Artificial Intelligence
COMBREX: Computational Bridges to Experiments
EC: Enzyme Commission
GAPDH: glyceraldehyde 3-phosphate dehydrogenase
GWAS: genome-wide association studies
KN: knowledge network
ML: Machine Learning
NCBI: National Center for Biotechnology Information
OMIM: Online Mendelian Inheritance in Man
STRING: Search Tool for the Retrieval of Interacting Genes/Proteins
